# Associations between Cyberbullying and School Bullying Victimization and Suicidal Ideation, Plans and Attempts among Canadian Schoolchildren

**DOI:** 10.1371/journal.pone.0102145

**Published:** 2014-07-30

**Authors:** Hugues Sampasa-Kanyinga, Paul Roumeliotis, Hao Xu

**Affiliations:** 1 Eastern Ontario Health Unit, Cornwall, Ontario, Canada; 2 Department of Pediatrics, McGill University, Montreal, Quebec, Canada; The University of Queensland, Australia

## Abstract

**Purpose:**

The negative effects of peer aggression on mental health are key issues for public health. The purpose of this study was to examine the associations between cyberbullying and school bullying victimization with suicidal ideation, plans and attempts among middle and high school students, and to test whether these relationships were mediated by reports of depression.

**Methods:**

Data for this study are from the 2011 Eastern Ontario Youth Risk Behaviour Survey, which is a cross-sectional regional school-based survey that was conducted among students in selected Grade 7 to 12 classes (1658 girls, 1341 boys; mean±SD age: 14.3±1.8 years).

**Results:**

Victims of cyberbullying and school bullying incurred a significantly higher risk of suicidal ideation (cyberbullying: crude odds ratio, 95% confidence interval  = 3.31, 2.16–5.07; school bullying: 3.48, 2.48–4.89), plans (cyberbullying: 2.79, 1.63–4.77; school bullying: 2.76, 2.20–3.45) and attempts (cyberbullying: 1.73, 1.26–2.38; school bullying: 1.64, 1.18–2.27) compared to those who had not encountered such threats. Results were similar when adjusting for sociodemographic characteristics, substance use, and sedentary activities. Mediation analyses indicated that depression fully mediated the relationship between cyberbullying victimization and each of the outcomes of suicidal ideation, plans and attempts. Depression also fully mediated the relationship between school bullying victimization and suicide attempts, but partially mediated the relationship between school bullying victimization and both suicidal ideation and plans.

**Conclusion:**

These findings support an association between both cyberbullying and school bullying victimization and risk of suicidal ideation, plans and attempts. The mediating role of depression on these links justifies the need for addressing depression among victims of both forms of bullying to prevent the risk of subsequent suicidal behaviours.

## Introduction

Suicide is a significant public health problem worldwide. It is the second leading cause of death for Canadian youth aged 10–24. Each year, on average, 294 youths die from suicide [1]. A recent report indicated that Eastern Ontario has a suicide attempt rate two times greater than the provincial average (6.78 vs. 2.96 per 1,000) [2]. Females aged 15 to 19 years in Eastern Ontario have a 50 per cent higher rate of suicide than the rest of the province. These alarming data support the crucial need for research data to understand the determinants of suicide and suicidal behaviour among children and adolescents. Experience of bullying victimization is one of many possible determinants of suicidal ideation and suicide-related behaviours.

Bullying – an aggressive behaviour that is intentional, repeated, and involves a power imbalance [3] – is a major public health concern. Despite the efforts of schools to prevent or stop bullying, bullying is still highly prevalent worldwide [4], [5]. Information and communication technology (ICT) has emerged as a recent vehicle for peer aggression worldwide. Cyberbullying has been defined as the use of email, cell phones, text messages, and Internet sites to threaten, harass, embarrass, or socially exclude [6], [7]. That is, cyberbullying provides to perpetrators the benefit of lack of face-to-face contact. It is more pervasive than traditional bullying, as it can happen anytime and anywhere. Consequently, bullying which usually takes place in school is now also occurring at home [8], [9], [10]. Among features that may distinguish cyberbullying from traditional bullying, the anonymity afforded to perpetrators and the limitless potential audience consisting of bystanders and observers are even more redoubtable [8], [11]. The inability for victims to have any control over acts of cyberbullying may result in feelings of powerlessness in the person being bullied [12]. As a result, the damage experienced in cyberbullying may be largely social and emotional in nature and is exacerbated by the intensity of the threats inflicted [12].

The growing number of cyberbullying victims over the past decade and the deleterious effects of cyberbullying on victims are of great concern. Several studies have shown that traditional bullying among youths is associated with depression, suicidal ideation and non-fatal suicidal behaviour [13], [14]. However, the psychological outcomes of cyberbullying remain inconsistent and unclear, probably because of its recent development. While some authors think that the consequences of cyberbullying tend to parallel those of traditional bullying [4], [11], others believe that cyberbullying may be even more psychologically distressing than regular school bullying [12]. An emerging body of research has begun to identify an association between cyberbullying and mental health problems. Several correlates have been identified among victims of cyberbullying, such as increased depression, suicidal ideation, and non-fatal suicidal behaviour (suicide attempts) [4], [15], [16].

**Figure 1:**
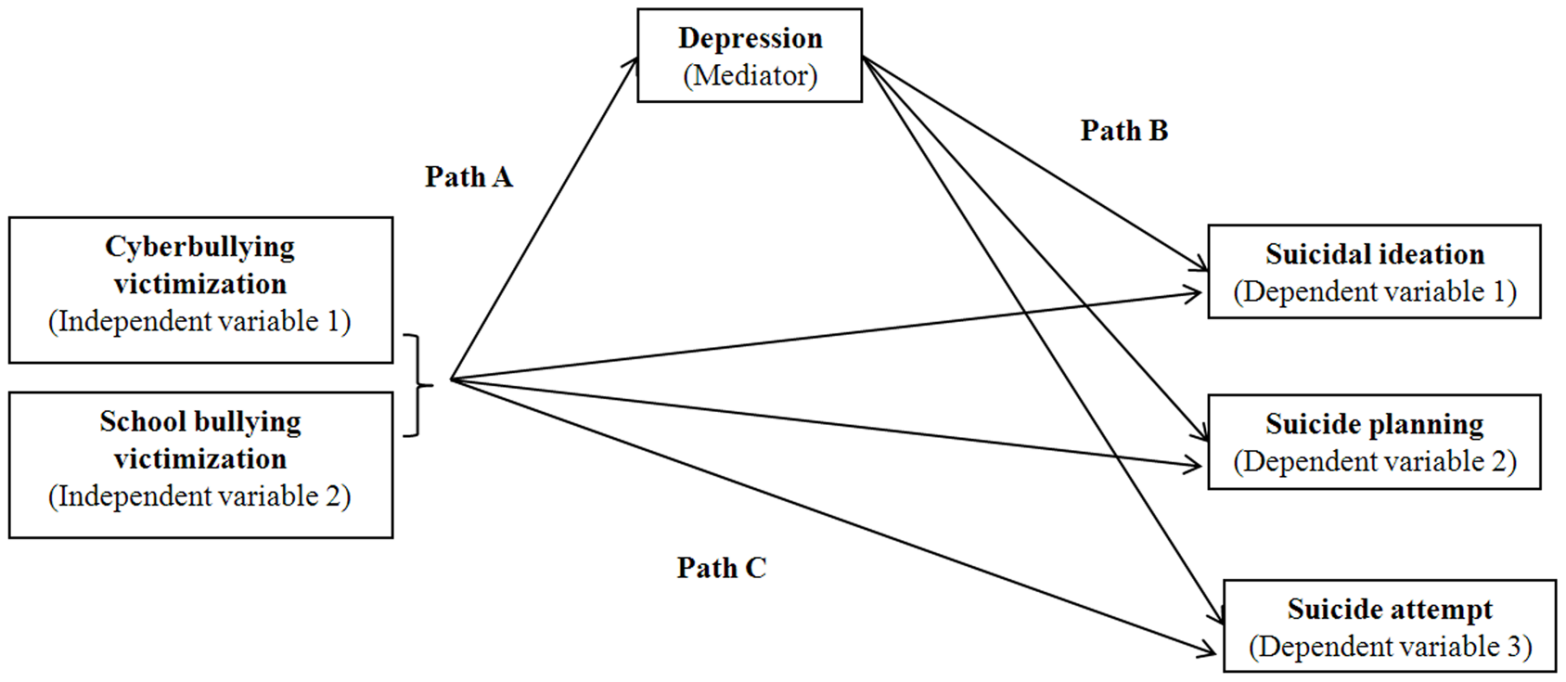
Mediational model for study of the relationship between cyberbullying and school bullying victimization and suicidal ideation, plans and attempts.

The psychological mechanisms underlying the association between bullying victimization and suicidal ideation and suicide-related behaviours are less well understood. According to Agnew's social psychological strain theory of deviance, strained social relationships and events pressure individuals into committing deviant acts [17]. Bullying is known to be a source of strain [18], [19]. It makes victims feel angry and frustrated, therefore putting them more at risk to engage in deviant behaviours. From this, it can be reasoned that bullying victims are at an increased risk of suicidal ideation, plans and attempts as coping responses to their victimization. Since depression is a well known risk factor for suicidal behaviour [20], it may therefore be accounted for in these causal chains as victims may first endure episodes of depression before progressing to suicidal ideation, plans and attempts (Figure 1). Based on the nature and intensity of threats and individual vulnerability of the victim, cyberbullying and school bullying may directly result in suicidal ideation, plans and attempts (Path C, Figure 1).

Bauman et al. [21] have recently documented the mediating role of depression on the association between bullying victimization and suicide attempts among American high school student girls using data from the 2009 Youth Risk Behaviour Survey (YRBS). However, their analyses were not adjusted for covariates and potential confounders reported in the literature to be associated with experience of bullying victimization and/or report of suicide attempts such as substance use [20], [22], [23], sedentary activities [24], and also importantly the amount of time spent on a computer [25], inasmuch as the experience of cyberbullying depends, at least in part, on the use of Internet. Thus, the independent effects (direct or indirect pathways) of bullying victimization on suicidal ideation and non-fatal suicidal behaviour controlling for other risks behaviours are not clear.

The purpose of the current study was to (1) examine the association between cyberbullying and school bullying victimization with suicidal ideation, plans and attempts among middle and high school students, as well as (2) test whether the presence of depression mediates these associations. We hypothesized that cyberbullying and school bullying victimization results in higher likelihood of suicidal ideation, plans and attempts, and that depression would mediate these relationships, while controlling for sociodemographic variables, substance use, sedentary activities, and the amount of time spent on the computer.

## Methods

The Eastern Ontario Youth Risk Behavior Survey (YRBS) is a regional cross-sectional school-based survey that has been conducted by the Eastern Ontario Health Unit (EOHU) for several years, and this paper uses the most recent data that was collected between November 2010 and March 2011 [26]. The survey was modeled after the Centers for Disease Control's (CDC) YRBS which monitors health-risk behaviours that contribute to death and disability among youths and adults [27].

### Ethical Statement

The study was approved by the Queen's University Health Sciences and Affiliated Teaching Hospitals Research Ethics Board. Signed consent forms from school principals and parents were verified prior to administering survey questionnaires.

### Participants

Youths in Grades 7 to 12 were the target population of the survey. Thus, all four school boards of the region were invited to join in the survey, and all schools within the boards that accepted the invitation were eligible for participation. Of 79 existing middle and high schools falling under EOHU jurisdiction in 2010, a total of 49 schools (62%) participated in the survey. This group of participating schools did not significantly differ from those that did not participate in the survey with respect to geographical location, school board (Catholic versus Public) or school type (middle versus high).

For each school that participated, a list of classes taking place during a certain time of the day was generated. The chosen time period varied by school and staff schedule. A random sample of two classes from each grade was selected in each school, unless only one grade was available for survey administration to all students who consented to participate. In this way, all students in Grades 7 to 12 had the same opportunity to be selected for participation and were not at risk of being selected to participate twice. The only exception was that students who had a spare period during the selected time slot were not eligible for participation. In the event of a teacher's refusal to participate, a second class for that grade was selected from the same time period. Students in selected classes were eligible to participate voluntarily and anonymously. The student response rate was 68%, for a total of 3,509 students aged 11 to 20 years (14.5±1.8 years), with 54.9% females. A School Liaison (Public Health Nurse or Health Educator Promoter) remained in the classroom while students responded to the survey to address questionnaire-related issues. If students did not understand a question, the Liaison would re-read the question without leading them in any particular direction.

### Measures

#### Bullying

Students were asked about school bullying and cyberbullying victimization in the past 12 months. The subject was introduced as follows: “The next questions are about bullying. Bullying is when one or more students tease, threaten, spread rumors about, hit, shove, or hurt another student over and over again. It is not bullying when two students of about the same strength or power argue or fight or tease each other in a friendly way.” School bullying and cyberbullying victimization were measured by the following questions: “During the past 12 months, have you ever been bullied or threatened by someone while on school property?” and “During the past 12 months, have you ever experienced cyberbullying, that is, being bullied by email, text messaging, instant messaging, social networking or another website?” Responses included “Yes” (coded as 1) or “No” (coded as 0).

#### Mental health

Items measuring mental health variables including depression, suicidal ideation, plans and attempts were taken from the Standard High School questionnaires of the CDC 2009 YRBS [27]. All yes-no response options were coded 1 for “Yes” and 0 for “No”. Depression was assessed by the following question: “During the past 12 months, did you ever feel so sad or hopeless almost every day for 2 weeks or more in a row that you stopped doing some usual activities?” (Yes or No). Suicidal ideation, plans and attempts were measured by the following questions, asked of all students: (1) “During the past 12 months, did you ever seriously consider attempting suicide?” (Yes or No); (2) “During the past 12 months, did you make a plan about how you would attempt suicide?” (Yes or No); and (3) “If you attempted suicide during the past 12 months, did any attempt result in injury, poisoning, or overdose that had to be treated by a doctor or nurse?” Response options included “I did not attempt suicide during the past 12 months” (coded as 0), Yes (equates to “attempted suicide that required medical attention”; coded as 1) or No (equates to “attempted suicide that did not require medical attention”; coded as 1). Combination of yes and no responses equates to “attempted suicide” (coded as 1).

#### Covariables

Demographic variables included age, sex (coded as 1 for girls and 0 for boys), and grade.Tobacco and alcohol use were measured by the following questions: “Have you ever smoked a whole cigarette?” and “Have you ever had at least one drink of alcohol, other than a few sips?” Responses included “Yes” (coded as 1) or “No” (coded as 0). Marijuana use was measured by two items: “During your life, how many times have you used marijuana?” Response options included: “0 times”, “1 to 2 times”, “3 to 5 times”, “6 to 9 times”, “10 to 19 times”, “20 or more times”. 0 times was coded 0 and rest of options were combined and coded “1”. Participants who responded having used marijuana at least once (coded as 1) answered to the following question: “what kind of marijuana user do you consider yourself?” Response options included: “A regular marijuana user” (coded as 1), “an occasional marijuana user” (coded as 2), “a non-user (but ever tried) (coded as 3). Sedentary activities were assessed by four items. In a typical day, how much time do you usually spend (1) using a computer, including playing computer games and using the Internet? (2) Playing video games, such as XBOX, Nintendo and/or PlayStation? (3) Watching television or movies? (4) Reading (not counting reading at school or at work)? For each item, participants were asked to provide an average number of hours and/or minutes spent per day.

Three categories were generated including: “less than 1 hour per day” (coded as 0), “one to three hours per day” (coded as 1) and “more than three hours per day” (coded as 2).

### Statistical analysis

Statistical analyses were conducted on individuals with no missing values for any of the variables studied. Of the 3509 students, 2999 (85.5% of the sample) were included in the analyses. With-and-without analyses showed that excluded missing data from the analyses did not have significant impact on the results. All data were analyzed with STATA (version 12.0, Stata Corp., College Station, Texas) using the svy commands to take account of the complex sampling design in variance estimates. Descriptive statistics on socio-demographic and risk behaviour correlates were calculated and stratified by cyberbullying and school bullying victimization status. Chi-square tests of significance were used to identify bivariate relationships between these characteristics and report of bullying victimization. There were no significant sex-by-cyberbullying victimization and sex-by-school bullying victimization interactions for any of the outcome variables of suicidal ideation, plans or attempts. As a result, all analyses were combined in order to maximize statistical power. Univariate and multivariate logistic regression analysis were performed to determine the associations between cyberbullying and school bullying victimization and each of the three outcomes of suicidal ideation, plans and attempts. Potential confounders included in the analysis were grade, gender, smoking, sedentary activities (including playing video games, such as XBOX, Nintendo and/or PlayStation, watching television or movies, and reading, not counting reading at school or at work), alcohol consumption, marijuana use, and the amount of time spent on the computer. All crude and adjusted odds ratios and 95% confidence interval (CI) are presented.

The mediating role of depression on the associations of bullying victimization (cyberbullying and school bullying) with suicidal ideation, plans and attempts was examined using the Baron and Kenny's approach [28] via a series of multivariate logistic models adjusted for grade, gender, smoking, sedentary activities, alcohol consumption, marijuana use, and time usually spent on the computer. The conditions necessary to demonstrate such a mediating relationship require: (1) a significant relationship between the independent (bullying victimization) and the dependent (suicidal ideation, plans and attempts) variables (*Path C*); (2) a significant association between independent (bullying victimization) and presumed mediator (depression) (*Path A*); (3) a significant association between presumed mediator (depression) and dependent (suicidal ideation, plans and attempts) variables (*Path B*). In the fourth and final regression model (4), after controlling the mediator (depression), the previously significant relationship between independent (bullying victimization) and dependent (suicidal ideation, plans and attempts) variables decreased or became non-significant. The Sobel test was used to statistically evaluate whether the indirect effect of bullying victimization on suicidal ideation, plans and attempts through depression was significant [29].

## Results

Table 1 reports the distribution of socio-demographic data and risk behaviour correlates for the whole sample and among cyberbullying and school bullying victims. Of the 2999 participants included in the analyses, 55.3% were female, and students in lower grades were oversampled. A total of 17.4% of students were victims of cyberbullying and 25.2% were victims of school bullying. There was a significant difference in the gender ratio of cyberbullying victims. Girls were twice as likely (22.3% vs. 11.4%, *p*<0.001) to experience cyberbullying victimization as boys (data not shown). Students who were in lower grades were more likely to be victims of school bullying. Participants who reported spending a lot of time on the computer or in other sedentary activities reported cyberbullying victimization more often than those who used computers for less time or spent less time in sedentary activities.

**Table 1:** **Sample Characteristics.**

Characteristics	Total	Cyberbullying victims	*p* value	School bullying victims	*p* value¶
Total	2999 (100)	522 (17.4)		757 (25.2)	
**Age**					
Mean age (SD)	14.3 (1.8)	14.5 (1.8)		14.2 (1.7)	
**Gender**			0.001		0.137
Male	1341 (44.7)	153 (29.3)		325 (42.9)	
Female	1658 (55.3)	369 (70.7)		432 (57.1)	
**Grade**			0.001		0.001
Grade 7	717 (23.9)	99 (19.0)		185 (24.4)	
Grade 8	616 (20.5)	97 (18.6)		171 (22.6)	
Grade 9	430 (14.3)	73 (14.0)		106 (14.0)	
Grade 10	439 (14.6)	103 (19.7)		129 (17.0)	
Grade 11	363 (12.1)	76 (14.6)		91 (12.0)	
Grade 12	434 (14.5)	74 (14.2)		75 (9.9)	
**Computer use** *			<0.001		0.001
<1 hour/day	902 (30.1)	98 (18.8)		206 (27.2)	
1 to 3 hours/day	1339 (44.6)	224 (42.9)		318 (42.0)	
>3 hours/day	758 (25.3)	200 (38.3)		233 (30.8)	
**Sedentary activities** †			<0.001		<0.001
<1 hour/day	82 (2.7)	11 (2.1)		21 (2.8)	
1 to 3 hours/day	470 (15.7)	52 (10.0)		91 (12.0)	
>3 hours/day	2447 (81.6)	459 (87.9)		645 (85.2)	
**Alcohol** ‡	1666 (55.6)	360 (69.0)	<0.001	455 (60.1)	0.004
**Smoking** §	500 (16.7)	144 (27.6)	<0.001	166 (21.9)	<0.001
**Marijuana** ||			<0.001		0.004
Regular user	87 (2.9)	24 (4.6)		32 (4.2)	
Occasional user	269 (9.0)	76 (14.6)		73 (9.6)	
Non-user (but ever tried)	211 (7.0)	47 (9.0)		67 (8.9)	
Never used	2432 (81.1)	375 (71.8)		585 (77.3)	
**Depression**	744 (24.8)	260 (49.8)	<0.001	311 (41.1)	<0.001

Data are shown as n (%).

*Time usually spent on the computer, including playing computer games and accessing the Internet on a typical day.

† Time usually spent on sedentary activities including playing video games, such as XBOX, Nintendo and/or PlayStation, watching television or movies, and reading, not counting reading at school or at work.

‡ Ever had at least one drink of alcohol, other than a few sips.

§ Ever smoked a whole cigarette.

|| Type of marijuana user.

¶ *p* value of association with cyberbullying victimization.

Table 2 presents the prevalence of suicidal ideation and suicide-related behaviours. Overall, the prevalence of suicidal ideation, plans and attempts was 10.5%, 10.7%, and 10.9%, respectively. Girls were more likely to report suicidal ideation and plan than boys. Among ideators, the conditional probability of making a plan was 64.6% and attempt was 49.7%. The probability of making an attempt among ideators with a plan was 58.3%, and 33.9% in ideators with no plan.

**Table 2:** **Prevalence of suicidal ideation and suicide-related behaviour among Canadian middle and high school students.**

	Ideation	Plan	Attempt	Plan among ideators	Attempt among ideators	Attempt among ideators with a plan	Attempt among ideators without a plan
**Total**	316 (10.5)	320 (10.7)	327 (10.9)	204 (64.6)	157 (49.7)	119 (58.3)	38 (33.9)
**Boys**	99 (7.4)	107 (8.0)	134 (10.0)	57 (57.6)	41 (41.4)	29 (50.9)	12 (28.6)
**Girls**	217 (13.1)	213 (12.5)	193 (11.6)	147 (67.7)	116 (53.5)	90 (61.2)	26 (37.1)
***p*** ** value** *	<0.001	<0.001	0.150	0.080	0.047	0.179	0.354

Data are shown as n (%).

*p value of difference between boys and girls.

### Association between bullying victimization and suicidal behaviour

Table 3 presents the crude associations between cyberbullying and school bullying victimization and suicidal ideation, plans and attempts. Victims of cyberbullying and school bullying incurred a significantly higher risk of suicidal ideation (cyberbullying: crude odds ratio, 95% confidence interval  = 3.31, 2.16–5.07; school bullying: 3.48, 2.48–4.89), plans (cyberbullying: 2.79, 1.63–4.77; school bullying: 2.76, 2.20–3.45) and attempts (cyberbullying: 1.73, 1.26–2.38; school bullying: 1.64, 1.18–2.27) compared to those who had not encountered such threats. Results were similar after controlling for grade, gender, sedentary activities, smoking, alcohol consumption, marijuana use, and the amount of time spent on a the computer (Table 4).

**Table 3:** **Associations between cyberbullying and school bullying victimization and suicidal ideation, plans and attempts.**

	Crude OR (95% CI)	P value
**Cyberbullying victimization**		
Suicidal ideation	3.31 (2.16–5.07)	0.001
Suicide planning	2.79 (1.63–4.77)	0.004
Suicide attempt	1.73 (1.26–2.38)	0.007
**School bullying victimization**		
Suicidal ideation	3.48 (2.48–4.89)	<0.001
Suicide planning	2.76 (2.20–3.45)	<0.001
Suicide attempt	1.64 (1.18–2.27)	0.012

**Table 4:** **Results of the mediational analyses on the relationship between cyberbullying and school bullying victimization and suicidal ideation, plans and attempts.**

	Path A		Path B		Path C				
	BV→Depression		Depression→Suicidal behaviour		BV→Suicidal behaviour				Sobel test
	OR (95% CI)	*P* value	OR (95% CI)	*P* value	OR_S−_ (95% CI)	P value	OR_S+_ (95% CI)	*P* value	(*P* value)
**Cyberbullying victimization**									
** Depression**	3.07 (2.49–3.80)	<0.001							
** Suicidal ideation**			9.72 (5.58–16.93)	<0.001	2.35 (1.50–3.70)	0.005	1.44 (0.84–2.45)	0.139	<0.001
** Suicide planning**			5.31 (3.73–7.57)	<0.001	2.06 (1.16–3.64)	0.023	1.39 (0.69–2.81)	0.276	<0.001
** Suicide attempt**			2.78 (1.79–4.31)	0.002	1.40 (1.04–1.90)	0.033	1.09 (0.79–1.51)	0.504	<0.001
**School bullying victimization**									
** Depression**	2.80 (2.40–3.26)	<0.001							
** Suicidal ideation**			9.05 (4.94–16.57)	<0.001	3.09 (2.18–4.39)	<0.001	2.16 (1.43–3.26)	0.005	<0.001
** Suicide planning**			5.03 (3.57–7.08)	<0.001	2.49 (1.97–3.17)	<0.001	1.86 (1.33–2.60)	0.005	<0.001
** Suicide attempt**			2.74 (1.77–4.23)	0.002	1.43 (1.04–1.98)	0.036	1.17 (0.83–1.65)	0.303	<0.001

*Note*. All models control for grade, gender, sedentary activities, smoking, alcohol, marijuana, and computer use.

BV: Bullying victimization.

OR_S−_: Odds ratio from regression model unadjusted for depression; OR_S+_: Odds ratio from regression model adjusted for depression.

### Mediation analysis

A series of multivariate logistic regression analyses were conducted and the results are summarized in Table 3. The regression coefficients of the three paths among the independent variables (cyberbullying victimization or school bullying), presumed mediator (depression), and each of the dependent variables (suicidal ideation, plans and attempts) were all significant. Entering depression into the equation of cyberbullying victimization rendered the main effect nonsignificant for all suicidal ideation, plans and attempts, whereas only the main effect of school bullying victimization on suicide attempts was rendered nonsignificant after controlling for depression.

Baron and Kenny's approach revealed therefore that the effects of cyberbullying victimization on suicidal ideation, plans and attempts were fully mediated by depression, and that depression also fully mediated the relationship between school bullying victimization and suicide attempts, but partially mediated the relationship between school bullying victimization and both suicidal ideation and plans. The Sobel test further showed evidence of the mediating role of depression in the relationship between cyberbullying and school bullying victimization and each of the suicidal ideation, plans and attempts outcomes among schoolchildren (p<0.001).

## Discussion

This regional school-based survey provided supporting evidence for the association among cyberbullying and school bullying victimization, suicidal ideation, plans and attempts among Canadian middle and high school students. Mediation analysis further indicated that depression fully mediated the relationship between cyberbullying victimization and each of the outcomes of suicidal ideation, plans and attempts. Depression also fully mediated the relationship between school bullying victimization and suicide attempts, but partially mediated the relationship between school bullying victimization and both suicidal ideation and plans. These findings corroborate and extend those from previous cross-sectional studies [4], [15], [16], and indicate that cyberbullying and school bullying victims are at risk of psychological distress, suicidal ideation and non-fatal suicidal behaviour. This evidence also supports the fact that bullying-related psychological distress and suicidal behaviour are associated with both online and off-line bullying victimization [4], [11]. However, since cyberbullying is an emerging phenomenon, lack of youth's awareness and psychological preparedness to deal with such issues might increase their vulnerability. Mishna et al. [30] reported a significant lack of knowledge regarding Internet safety among youths. Enhancing awareness among schoolchildren is therefore a crucial step towards preventing cyberbullying victimization. This could be tackled by parents and schools discussing Internet safety and cyberbullying with children. On the other hand, there is a need to strengthen ongoing bullying prevention to make school environment a safe and happy place.

Our findings on the mediating role of depression on the relationship between bullying victimization and non-fatal suicidal behaviour are congruent with those of Bauman et al. [21] and justify the need for addressing depression among victims of cyberbullying and school bullying to prevent the risk for subsequent suicidal behaviour. The mediating role of depression suggests that cyberbullying and school bullying victimization among schoolchildren may lead to elevated depressive symptoms, resulting in more suicidal ideation, plans and attempts. However, the partial mediating role of depression in the relationships between school bullying victimization and both suicidal ideation and plans suggest that factors other than psychological distress may also play a role as mediators in these etiological relationships. Indeed, there are many other factors that can contribute to teenagers' suicidal behaviour (e.g. family violence, sexual orientation, physical and sexual abuse, interpersonal losses, etc.) [20]. It is crucial to provide suicide prevention training to teachers and parents to help them identify symptoms or changes in behaviour related to depression and suicide among children and adolescents. Besides, schoolchildren should be encouraged to seek support from peers and family when experiencing bullying victimization. This has been reported to provide significant buffering effects on depressive symptoms [31].

The relationship between bullying victimization and depression is reciprocal. Bullying victimization can cause depression and depressive symptoms may place some youths at increased risk for victimization. Longitudinal studies have shown such reciprocal effects. Gamez-Guadix et al. [22] recently reported a such association between cyberbullying victimization and depression. However, Bond et al. [32] demonstrated that the path from victimization to depression was stronger than the path from depression to victimization; which supports our hypothesized mediational pathway. Given the cross-sectional nature of the current study, we cannot rule out the relation from depression to bullying victimization.

The prevalence of cyberbullying (17.4%) and school bullying (25.2%) documented in the current study are comparable to those recently reported in Canada [33], [34] and elsewhere [4] using similar definitions and time frames to assess cyberbullying and school bullying victimization, and the same age groups (grade). These substantial proportions indicate that school bullying is still prevalent among middle and high school students, and that cyberbullying also largely occurs within this population. This supports the need for further actions and attention to protect children and adolescents. Results revealed gender differences in report of cyberbullying victimization, but not school bullying victimization. Girls were twice as likely to experience cyberbullying victimization as boys. These results are congruent with several other studies that documented a higher prevalence of cyberbullying victimization among girls [35], [36], [37]. This may be due to the fact that cyberbullying is text-based, and girls communicate more often using text messaging and email than boys [38].

Not surprisingly, our findings also indicated that the longer the time spent on the computer, the greater the likelihood of cyberbullying victimization. Similar results have been reported by Hinduja and Patchin [25] who observed that time spent on-line and computer proficiency were related to cyberbullying behaviour. Augmented time spent on Internet heightens the likelihood of experiencing cyberbullying. Given the prominent place of the Internet in today's lifestyle, especially among children, banning it is quite impossible and may not be a helpful measure against cyberbullying. Instead, placing limits on time spent on the computer may help decrease such threats. Furthermore, it is important for parents and schools to learn how to keep children safe online.

The Eastern Ontario region is recognized to have higher rate of suicide in comparison with the provincial average [2]; therefore, we expected to have higher proportion of students who endorse suicidal ideation and attempts during the past 12 months. Indeed, our rates were quite high, especially for suicide attempt. Results indicated that nearly two-thirds of participants who seriously considered suicide made a plan of how they would attempt suicide, and three out of five of those who both seriously considered suicide and made a plan have attempted suicide. This may be related to the rurality and remoteness of the region. In fact, nearly half of the population of the Eastern Ontario region lives in rural areas; and, rurality and remoteness are well known to be associated with greater suicide and suicidal behaviour for both adults and adolescents [39].The prevalence of suicidal ideation within the last 12 months documented in our sample (10.2%) was comparable to those reported in the neighboring city of Ottawa (12%) [33] and the province of Ontario (10%) [34], but lower than those reported by the CDC's 2009 YRBS (13.8%) and Bauman et al. (2013) (17%). However, our prevalence of suicide attempt (10.9%) is higher than those reported at the provincial level (3%) [34] and by the CDC's 2009 YRBS (6.3%), but comparable to rate of Bauman et al. (2013) (10%). Differences in age demographics (grades) may explain the observed variations with the USA data. Suicidality data collected from the CDC are based on high school students from grades 9–12. In contrast, our sample encompasses youth from grades 7–12, and students in lower grades were oversampled. The questions related to suicide may have been difficult for the younger students to grasp. The prevalence of suicidal ideation and attempts reported herein are likely to be underestimates given the fact that suicidal behaviour generally increases with age [20].

This study has several limitations that need to be addressed. First, the questionnaires administered in our survey were not specifically designed to assess cyberbullying, school bullying or suicide. For example, the item that measured time spent using a computer may not truly reflect the amount of time spent using the Internet because computer game playing was also included, whereas cyberbullying is specifically framed on the Internet use. Furthermore, as cell phones are becoming more technologically advanced, and many of them allow access to the Internet, children may prefer using cell phones rather than computers. As such, we have most likely underestimated the amount of time spent using the Internet. Second, the cross-sectional nature of the data precludes evaluation of temporality and causality of the observed relationship between both forms of bullying victimization and each outcome of suicidal ideation, plans and attempts. Future research with longitudinal data will be important for establishing temporal sequence. A third limitation of our study concerns the reliability of the data gathered. Despite demonstrated test-retest reliability of the YRBS among students from across these grades [40], potential reliability issues might be associated with self-reporting data from these age groups. Finally, even if our survey was designed to represent the population of Eastern Ontario, the response rates to the survey may not warrant the generalizability of the findings. The oversampling of students in lower grades, however, may have likely reduced the strength of the estimates.

Despite these limitations, this study addresses gaps in the literature and is among the first to examine the mediational pathways on the association among cyberbullying and school bullying victimization and suicidal ideation, plans and attempts. Our findings indicated the presence of strong and significant associations between both forms of bullying victimization and suicidal ideation, plans and attempts, as well as the full mediating role of depression. These results support the need to address depression among victims of cyber and school bullying to help prevent the risk for subsequent suicidal behaviour. Parents and teachers need to pay more attention to and support youths with respect to cyberbullying victimization. Given the rapid advance of ICT, the number of cyberbullying victims will increase, giving rise to a need for safety measures to be implemented to make the Internet safer for children. More research is needed with longitudinal designs in order to examine the relationship between cyberbullying and school bullying victimization, depression and suicidal behaviour among children and adolescents.
